# Numerical Modeling Design for the Hybrid Additive Manufacturing of Laser Directed Energy Deposition and Shot Peening Forming Fe–Cr–Ni–B–Si Alloy

**DOI:** 10.3390/ma13214877

**Published:** 2020-10-30

**Authors:** Xiaoyu Zhang, Dichen Li, Weijun Zhu

**Affiliations:** 1 State Key Laboratory of Manufacturing Systems Engineering, Xi’an Jiaotong University, Xi’an 710049, China; zhxy6396@stu.xjtu.edu.cn; 2 School of Mechanical Engineering & Automation, Beijing University of Aeronautics & Astronautics, Beijing 100191, China; zhuweijun@buaa.edu.cn

**Keywords:** hybrid additive manufacturing, laser directed energy deposition, shot peening, process modeling, position of injection point

## Abstract

Hybrid additive manufacturing is of great significance to make up for the deficiency of the metal forming process; it has been one of the main trends of additive manufacturing in recent years. The hybrid process of laser directed energy deposition (laser DED) and shot peening is a new technology combining the principles of surface strengthening and additive manufacturing, whose difficulty is to reduce the interaction between the two processes. In this paper, a new model with a discrete phase and fluid–solid interaction method is established, and the location of the shot peening point in the hybrid process is optimized. The distributions of the temperature field and powder trajectory were researched and experiments were carried out with the optimized parameters to verify simulation results. It was found that the temperature field and the powder trajectory partly change, and the optimized injection point is located in the stress relaxation zone of the material. The densities and surface residual stresses of samples were improved, and the density increased by 8.83%. The surface stress changed from tensile stress to compressive stress, and the introduced compressive stress by shot peening was 2.26 times the tensile stress produced by laser directed energy deposition.

## 1. Introduction

Metal additive manufacturing technology is based on the principle of “separating and accumulation,” using a laser, an electron beam, an arc beam or another high-energy beam as a heat source and taking materials of metal wire and powder to melt and solidify layer by layer to form parts [1,2,3]. At present, metal additive manufacturing technology has developed rapidly in the fields of aerospace, bio-manufacturing, automobiles, military and so on [4,5,6]. Hybrid metal additive manufacturing technology, as a method to solve the problems of internal defects [7,8,9,10], forming stress and low precision in metal additive manufacturing, has become a research focus in recent years. The hybrid manufacturing process of laser DED and shot peening is a field that has not been extensively and deeply explored and studied, which can effectively improve the density, stress state and surface roughness, and make up for the deficiency of metal additive manufacturing technology [11,12]. Combining the principle of additive manufacturing with the peening strengthening, the surface strengthening technology is transformed into the whole strengthening technology by layer and layer enhancing. However, the position of the shot peening point in the hybrid process is a difficult problem, which restricts the development and application of it. The convergence of powder in laser DED and the forming precision and quality will be affected when the shots are sprayed over a short distance. On the other hand, when shots spray over a long distance, the temperature of the material at the injection point decreases, and the material can have a work hardening effect, affecting the forming quality of the hybrid process. Therefore, location of the shot peening point is one of the key parameters of hybrid manufacturing of laser cladding deposition and shot peening.

As the temperature field near the laser cladding pool changes rapidly and is complex in the hybrid process [13,14,15,16], and the speed of peening powder is high, it is difficult to detect the exact temperature of the area of shot peening. With the development of numerical calculations and computer science, a simulation can provide scientific and reasonable guidance for the actual process, especially in the field of laser DED. In recent years, many researchers have carried out a lot of research on process simulation. Ibarra-Medina established the computational fluid dynamics (CFD) model of laser DED and the interactions between the laser, powder flow and substrate were studied [17]. Qian L built a CFD model of gas-powder flow for the design of powder feeding nozzles in cladding, and studied the influences of geometry of nozzle outlet, powder properties and powder feeding parameters on characteristics of powder flow [18]. Nie P used CFD method, physical energy function and finite element method to simulate the laser cladding process of nickel-based high-temperature alloy, and studied the relationship between solidification structure and process parameters [19]. Tabernero I used the CFD method to simulate the process of powder feeding in laser DED, and studied the influences of particle size distribution and feeding rate [20]. Tsai H L carried out a turbulent two-phase CFD simulation of gas-powder flow in the laser DED, studied the influence of the change of powder feeding injection angle on powder convergence and assisted in locating the laser spot [21]. In the previous work, a lot of researchers established CFD models of laser DED to optimize design and parameters. This method is suitable for predicting the heat change and characteristics of gas-powder flow during laser DED, especially suitable for the situation of high speed powder in shot peening and a complex temperature field in laser DED and for the research of the movement of powder and the influence of gas flow on the temperature field.

Based on the hybrid process of laser DED and shot peening, an interactive model for analyzing temperature field, shot peening and powder trajectory is established in this paper. Aiming at shot peening position, one of the key parameters in the hybrid process, the distance of shot peening, is optimized by the method of comprehensive consideration of spraying angle and temperature field. The influences of gas flow on the cladding temperature field and powder convergence property in the hybrid process are studied, and the optimized process parameters were verified by experiments. The hybrid process can be realized.

## 2. Numerical Model

### 2.1. Calculation Scheme

**Figure 1:**
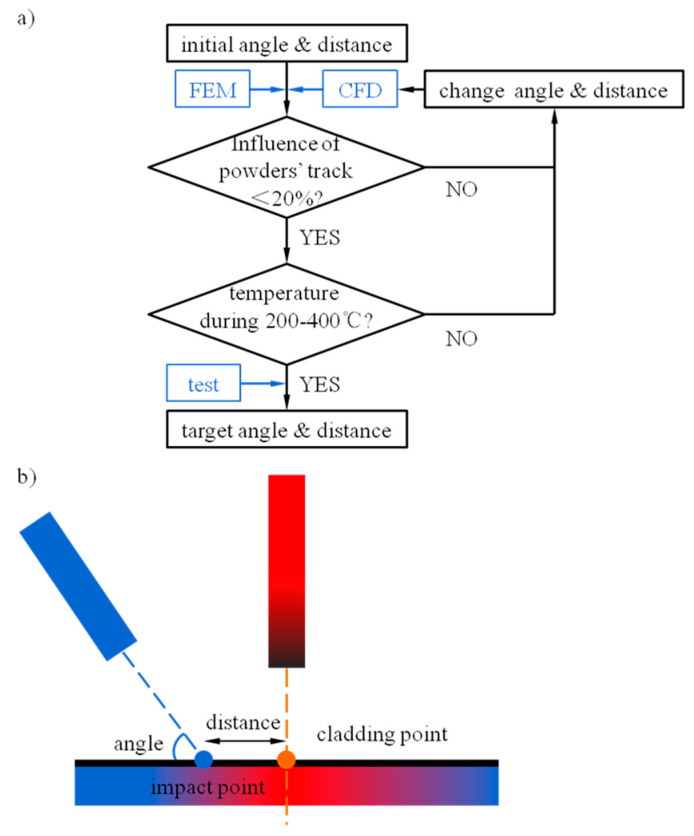
The calculation scheme and schematic diagram of the hybrid process. (**a**) The calculation scheme and (**b**) schematic diagram of the hybrid process.

In this paper, a CFD model with the discrete phase model and fluid–solid coupling method is used, and the hybrid process of laser DED and shot peening is simulated. The simulation process was performed by the commercial software Fluent. The influences of gas flow and powder were detected under the optimal parameters, and the temperature field in the impact area of high-speed shot peening was also detected to determine whether it is located in the temperature range of stress relaxation. The angle and distance of shot peening were changed in the calculation scheme. The optimal position was approached by the method of dichotomy and the optimal parameter of shot peening in the hybrid process was determined. The simulation results were verified by experiments. The calculation scheme and schematic diagram of the hybrid process are shown in Figure 1, where (a) is the calculation scheme and (b) is the schematic diagram of the hybrid process.

### 2.2. Flow Analysis

The flow field and powder trajectory are simulated by establishing a two-dimensional steady-state model. The properties of a fluid change violently in the hybrid process, such as the momentum, heat, mass and average Reynolds number reached in the turbulent process. Therefore, the realizable *k*-*ε* turbulence model is adopted in this paper, which has more adaptability in the calculation of high speed jets compared with the standard model and Re-normalization group (RNG) model [22,23]. The gas in the model is assumed to be an ideal gas. The flow is constant and does not change with time, and the flow law meets the ideal gas law. In addition, Navier–Stokes equations based on mass and momentum are used to calculate the fluid dynamics in this model shown in Equations (1) and (2), and the influence of turbulence velocity fluctuation is taken into account in the calculation [24].

(1) $$\frac{\partial \rho }{\partial t}+\nabla \cdot (\rho \vec{v})=s_{m}$$

(2) $$\frac{\partial }{\partial t}(\rho \vec{v})+\nabla \cdot (\rho \vec{v}\vec{v})=-\nabla p+\nabla \cdot (\bar{\bar{\tau }})+\rho \vec{g}+\vec{F}$$

where *ρ* is the fluid density, $\vec{v}$ is the fluid phase velocity, *s_m_* is the added mass of the system due to the flow of discrete phases, p is the static pressure, $\bar{\bar{\tau }}$ is the stress tensor, $p\vec{g}$ is a body force due to gravity and $\vec{F}$ is any other body force term produced in the interaction with the second phase, or terms due to the functional requirements of the model.

The analysis of the gas-powder model has the characteristics of two-phase coupling [25]. The heat transfer coupling between the gas phase and the particles and the influence of the gas flow resistance on particles should be considered in the model of the hybrid process. The behavior of the suspended particles depends on the characteristics of the powder and the gas flow. Many methods have been proposed in recent years, but due to the complexity of gas flow and particle movement, the prediction model still has great uncertainty. To consider the influence of turbulence on particle dispersion, a discrete random walk model was used to track a single particle, and the trajectory equation of a single particle was integrated to predict the turbulence dispersion of particles. If enough trajectories are calculated, the statistical data of the spread of the particle stream in the turbulence model can be obtained, and the distribution range of shot peening can be also obtained. In this paper, the motion trajectory of powder is simulated and predicted by using the discrete phase model in the Lagrangian coordinate system. The force balance equation established for particles in the Cartesian coordinate system is shown in Equation (3), which contains additional force and is important in some special cases.

(3) $$\frac{du_{p}}{dt}=F_{D}(u_{f}-u_{p})+\frac{g_{i}(\rho_{p}-\rho_{f})}{\rho_{p}}+F_{i}$$

where *u_p_* is particle velocity, *u_f_* is the velocity of the fluid, *F_D_*(*u_f_* − *u_p_*) is the drag force per unit particle mass, *g_i_* is the gravity coefficient, *ρ_p_* is the particle density, *ρ_f_* is fluid density, *Fi* is an additional acceleration term and

(4) $$F_{D}=\frac{18\mu }{\rho_{p}d_{p}^{2}}\frac{C_{D}Re}{24}$$

where *Re* is the Reynolds number, *C_D_* is the drag coefficient, *μ* is the molecular viscosity of the fluid and *d_p_* is the particle diameter.

### 2.3. Temperature Analysis

The heat source in the hybrid process is generated by laser, and the heat conduction process mainly consists of two parts: the heat transfer of gas flow generated by shot peening and the heat transfer inside the solid. Since the laser has high energy density and a stabilized output characteristic, it is assumed that the temperature of the central molten pool is not affected by the external environment and remains constant. For the energy distribution of the laser used in the equipment with a near flat distribution, the laser source is introduced by setting 1670 K which is the melting point of the material as the boundary temperature of molten pool. It is supposed that laser energy melts the material completely. For the velocity of shot peening when the powder is high enough, the shielding effect of the particles on the laser is omitted. It is an important part of the heat transfer model at the interface between gas and solid phases. The heat transfer amounts of the coupling boundary regarding fluid and solid are shown as Equations (5) and (6).

(5) $$q\prime \prime {=h}_{f}(T_{w}-T_{f})+{q\prime \prime }_{rad}$$

where *h_f_* is the convective heat transfer coefficient, *T_w_* is the wall temperature, *T_f_* is the fluid temperature and $q_{rad}^{\prime \prime }$ is the radiation heat transfer.

(6) $$q\prime \prime =\frac{K_{s}}{\Delta n}(T_{w}-T_{s})+q_{rad}\prime \prime$$

where *K_s_* is the heat transfer coefficient, ∆n is the normal vector and *T_s_* is the solid temperature.

In the solid, only heat conduction is considered. As shown in Equation (7), the heat conduction equation is established:

(7) $$\frac{\partial }{\partial t}(\rho h)+\nabla \cdot (v\rho h)=\nabla \cdot (k\nabla T)+S_{h}$$

where *v* represents the velocity of the solid due to rotation or translation, ∇⋅(k∇T) is the heat flow caused by heat conduction and *S_h_* represents the internal heat source in the solid.

The material used in this paper is Fe–Cr–Ni–B–Si alloy, a kind of austenitic stainless steel. The thermal conductivities of austenitic stainless steel are close to each other, and thermal conductivity of 08Kh18N10T austenitic steel is used in heat transfer process, whose thermal conductivity is approximately close to material used in this paper. Its parameters are shown in Table 1 [26].

### 2.4. Model Design and Boundary Conditions

**Figure 2:**
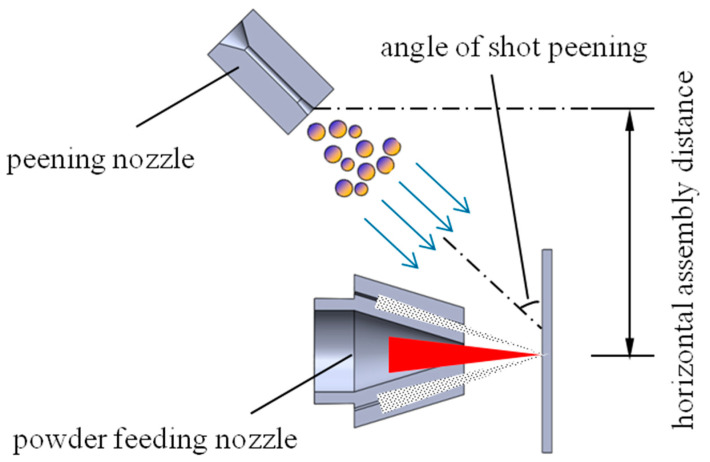
The schematic diagram of the hybrid equipment.

A 2D axisymmetric model was established by combining the powder feeding channel, a shot peening nozzle flow channel, forming space and substrate in the actual equipment of the hybrid process of laser DED and shot peening. The schematic diagram of laser cladding and shot peening hybrid equipment is shown in Figure 2. To ensure that the shot peening position is as close to the molten pool as possible, the shot peening nozzle should be assembled as close to the cladding nozzle as possible. Due to the limitations of assembly space and nozzle geometry size, the closest horizontal distance between the shot peening nozzle and the laser cladding nozzle is 75 mm, and the position of the shot peening can be changed by injection angle of the nozzle. The shot peening nozzle was a Venturi nozzle, with an inlet diameter of 10 mm, an inlet angle of the contraction part of 60°, a throat tube diameter of 2 mm, an outlet angle of the expansion part of 10° and an inner diameter of the powder feeding tube of the laser cladding nozzle of 1 mm. The position of the impact point varies within the range of 5–10 mm from the cladding point.

**Figure 3:**
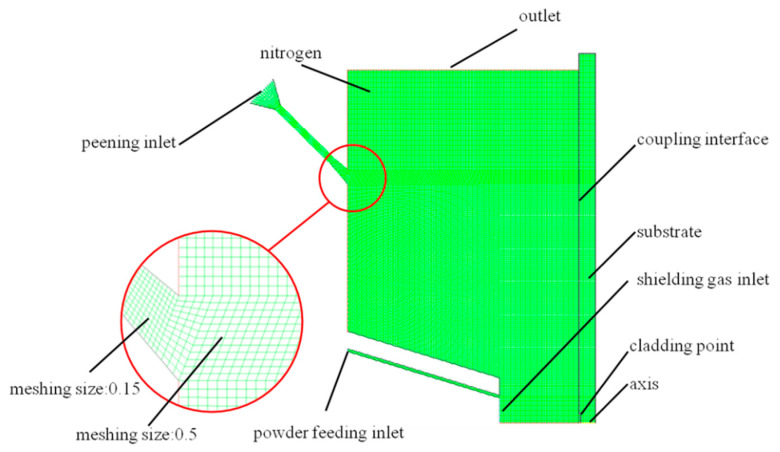
The mesh generation diagram.

An axisymmetric simplified calculation method is adopted for the model mesh generation. The center of laser is used as the symmetry axis to maximize the operation efficiency. Due to the almost axisymmetric temperature distribution of molten pool and the quasi axisymmetric powder convergence in previous results of 3D models with imperfect mesh, we adopted a 2D axisymmetric model to get more accurate results efficiently. The mesh generation diagram is shown in Figure 3. The quadrilateral unstructured meshes were adopted, and the methods of local mesh refining are introduced here. In order to improve the accuracy of calculation, meshes are refined in the places where gas flow changes dramatically, such as the inner flow passage of shot peening nozzle and powder feeding nozzle, and the mesh size is 0.15 mm. In the forming space, the mesh size of the area with weak gas flow is 0.5 mm to improve the efficiency of operation. The substrate is set as a solid mesh. The interaction interface with the gas is set as a coupling interface to study the influence of shot peening gas flow on the temperature field in the hybrid process. The wall types in the model are reflective, in which the inner flow passage of the shot peening nozzle is ceramic material, and the inner wall of the powder feeding tube is copper material. The pressure at the shot peening inlet is 0.4 MPa; the pressure at the powder feeding tube and the shielding gas inlet is 0.3 MPa. The initial boundary conditions of the model are shown in Table 2.

### 2.5. Experimental Scheme and Measuring Equipment

The temperature field and powder trajectory of the hybrid process under optimized parameters were verified by experiments according to the simulation results. An infrared thermal imager was used to detect the temperature field in the hybrid process. After comparing the actual temperature curve with the simulation result, the feasibility of the process and the reason for the simulation error were analyzed. The infrared thermal imager equipment type was X6520sc (Flir, Wilsonville, OR, USA). The pixel spacing of the equipment was 15 μm. The test of process interferences is mainly to observe the change of powder trajectory in the hybrid process and analyze its influence on the forming workpiece. Finally, the simulation was used to optimize the process parameters for the forming experiment. Stress, density, hardness and roughness of samples were measured. The stress testing equipment was an X-ray stress measuring instrument, the type of which was X-350A (Handan ST Institute, Handan, China). The emissivity was 0.35 [27]. The average stress of 3 points on the surface along the direction of cladding was measured. The density was measured by drainage method. The roughness detection equipment was a roughness measuring instrument, whose type was TR300 (Shanghai optical instrument factory, Shanghai, China), and 3 lines were measured. Hardness was measured by microhardness tester whose type was HXD-2000TMSC/LCD (Taiming, Changzhou, China). Hardnesses of 10 points on a cross-section were measured. The equipment made by Xi’an Jiaotong University (Xi’an, China) in the experiment was a hybrid of laser DED and shot peening. The laser power was 1054 W, the spot size was 3 mm, the scanning speed was 40 mm/s, the layer thickness was 0.3 mm, 20 layers were formed and the powder feeding quantity was 15.94 g/min. Fe–Cr–Ni–B–Si alloy powder (a type of austenitic stainless steel) was used in the experiment, whose components are shown in Table 3. Powder diameter was approximately 100 μm. The parameters of shot peening process were optimized by simulation.

## 3. Results

### 3.1. Simulation Results

#### 3.1.1. Change of Temperature Field and Determination of Injection Point

**Figure 4:**
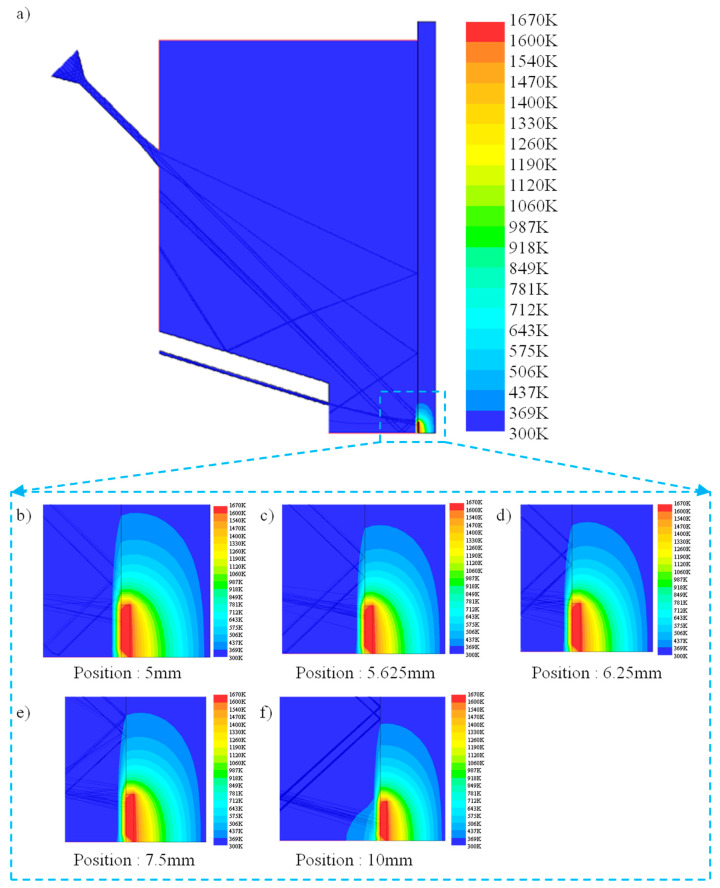
Temperature field distribution and powder trajectory near the injection point. (**a**) Overall schematic diagram and (**b**–**f**) particle track and temperature field changes at different shot positions.

**Figure 5:**
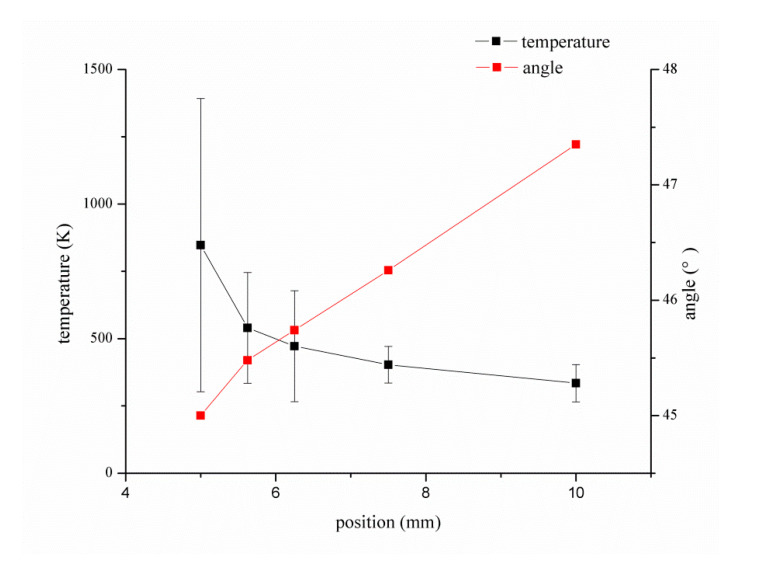
Temperature and angle variations at injection point.

The closer the shot peening point is to the cladding point, the higher the temperature of the material is, and the stress is in a relaxed state, which makes it easier to eliminate the residual tensile stress generated during the cladding process. However, it is easier to influence the powder convergence during the laser cladding process, and at the same time, the shot peening injection angle is reduced, which also restricts the shot peening effect. Therefore, considering the temperature field distribution of injection distance and injection angle, the optimum shot peening point of the hybrid process is determined. Figure 4 shows the temperature field distribution near the shot peening injection point, and the following figure shows the particle track and temperature field changes near the molten pool. According to the principle of dichotomy, the stress relaxation area of the material is approached step by step. When the injection point position is 10 mm from the cladding point, the temperature of the material at injection point position is low. When the shot peening point gets close to the cladding point gradually, the influence of the gas flow decreases, and the temperature field diffused by the cladding point on the substrate weakens. We recorded the temperature interval of the material and the injection angle near the shot peening point. Figure 5 shows the temperature interval and angle change diagram near the injection point, and the bar represents the temperature range of the shot peening area with different injection angles. When the injection point is closer to the cladding region, the longer the temperature interval span is, the more drastic the temperature change is. The temperature field is mainly located near the injection point with an injection angle of 45°. According to the intersection position of the two curves of injection angle and temperature, the optimal injection point was determined to be 6.04 mm away from the cladding point, and the injection angle was 45.65°. Due to the limitation of assembly accuracy, the injection point used in the experiment was 6 mm from the cladding point and the injection angle was 45.6°. The reference point was the central axis of the shot peening nozzle. Additionally, the cladding point was set at the center of laser indicator light.

**Figure 6:**
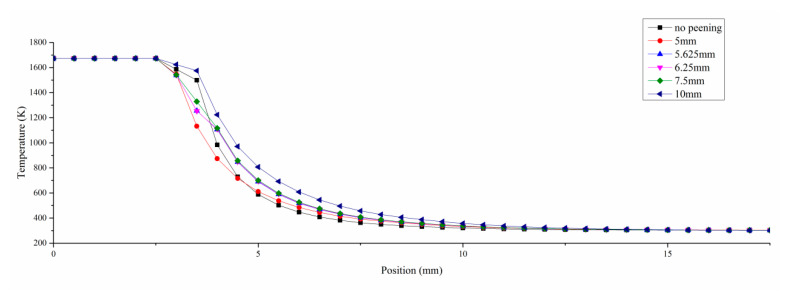
The temperature changes at different injection point positions.

**Figure 7:**
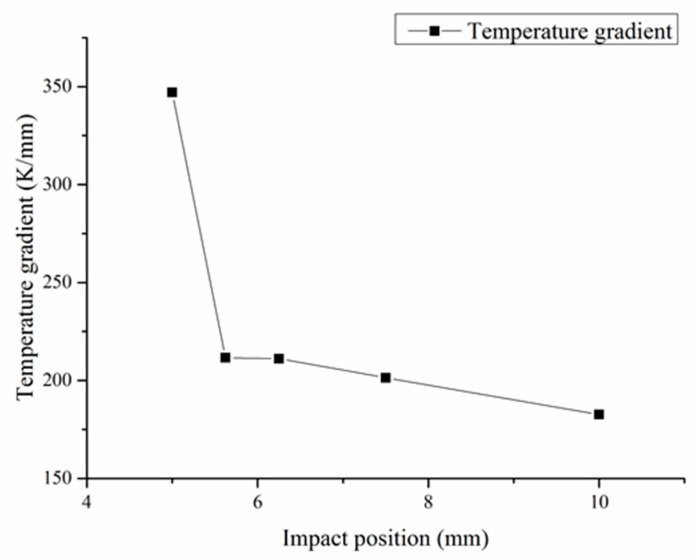
The temperature gradient changes at different injection point positions.

Figure 6 shows the temperature changes at different injection point positions, and Figure 7 shows the temperature gradient changes at different injection point positions. Due to the convective heat transfer by gas flow, the temperature field in laser DED changed to some extent. Compared with the single cladding process, the temperature decreases faster in the high temperature area and slower in the low temperature area during the hybrid process. At the same time, the temperature changes with the position of the shot peening point. As the shot peening point is far away from the molten pool, the temperature gradient gradually decreases and the temperature reduction rate slows down. When the shot peening point is located within 6 mm from the center of the molten pool, the temperature gradient changes dramatically. When the shot peening point is located 10 mm away from the center of the molten pool, the temperature around the molten pool decreases slowly in the hybrid process. With the intense and complex evolution process of metal melting and solidification, when affected by the gas flow, it presents a bidirectional heat dissipation trend from the jet point as the center, toward and away from the molten pool. Compared with the low temperature region, the influence of gas flow on the high temperature region is greater. Different temperature areas of the material show different change laws. The temperature in the high temperature area drops rapidly. In particular, when the injection point is gradually further from the center of the molten pool, the temperature in the low temperature area rises somewhat due to heat flow diffusion by gas flow.

#### 3.1.2. Process Interference Analysis

**Figure 8:**
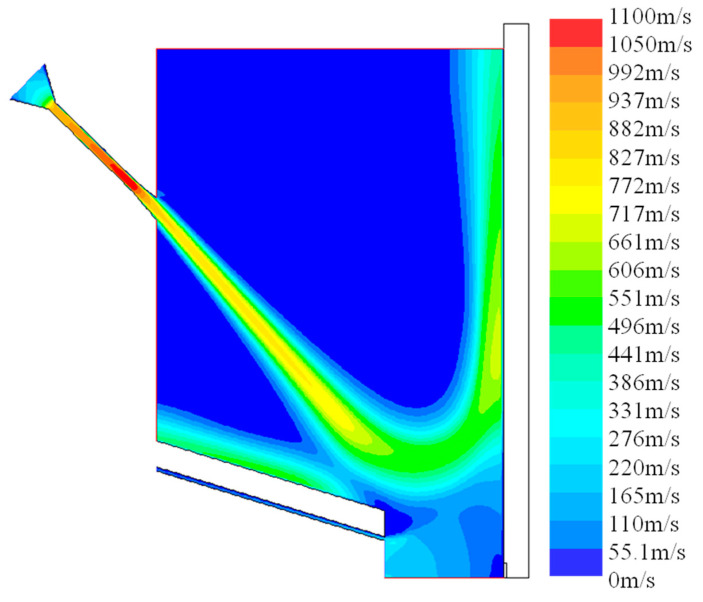
Cloud diagram of gas flow velocity under optimal parameters.

**Figure 9:**
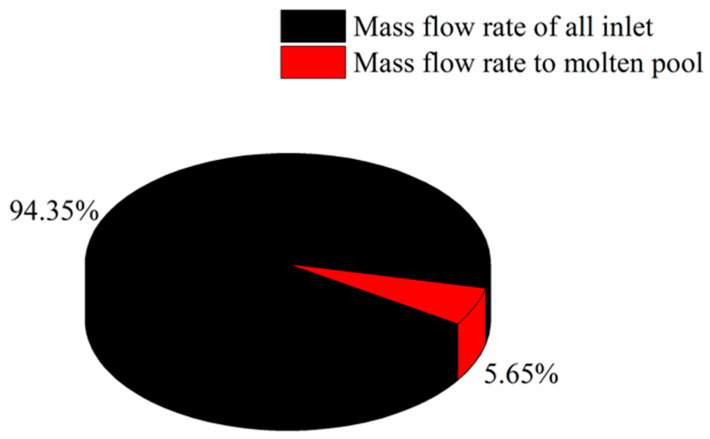
Mass flow rate statistics in the hybrid process.

The interference between shot peening and laser DED is inevitable in the hybrid process, which is mainly manifested by the introduction of cold gas flow and the weakening of powder convergence. The results of flow field distribution and powder trajectory under the optimal injection location were analyzed to reduce the forming material defects in laser DED. Figure 8 shows the cloud diagram of gas flow velocity under optimal parameters, and Figure 9 shows mass flow rate statistics in the hybrid process. As shown in the figure, a large amount of gas flow escapes from the exit of the shot peening nozzle. Before reaching the substrate, due to the influence of powder feeding gas, protective gas and hot pressure near the molten pool, the gas flow flows away from the cladding point, and a small amount of gas flow flows to the forming area. The gas flow to the forming area accounts for about 5.65% of the total inlet gas flow, and the influence of gas flow is relatively less than 20%.

**Figure 10:**
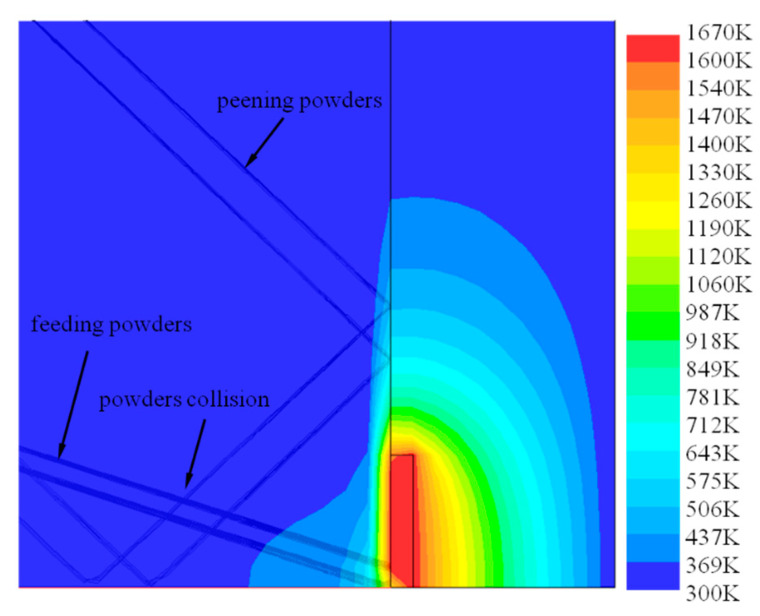
The distribution of the powder flow and the temperature field under the optimal injection point.

**Figure 11:**
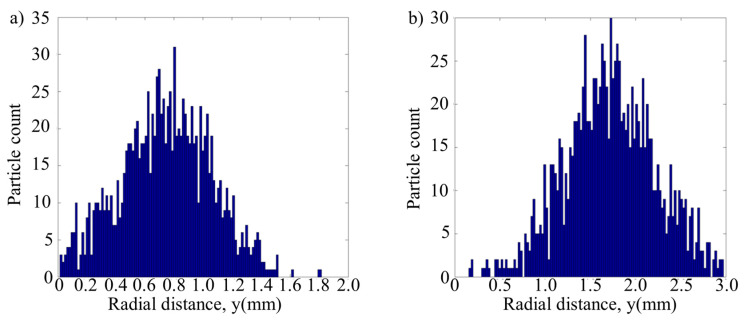
Statistical chart of powder quantity. (**a**) Powder quantity in laser DED and (**b**) powder quantity in the hybrid process.

**Figure 12:**
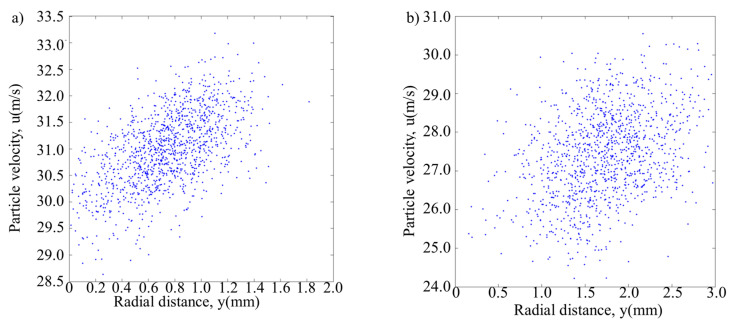
The distribution diagram of powder velocity. (**a**) Powder velocity in laser DED and (**b**) powder velocity in the hybrid process.

Figure 10 shows the distribution of powder flow and the temperature field under the optimal injection point in the hybrid process. It can be seen in the figure that the temperature near the injection point is about 575–712 K (302–439 °C), which is basically located in the stress relaxation zone of austenitic stainless steel [26]. Shot peening can have a better effect on the material in a state of stress relaxation. The shot peening powders and the forming powders collide near the substrate, the forming powders still keep good convergence and the forming powders can still enter the molten pool to realize the cladding process. Figure 11 shows the statistical chart of powder quantity at different positions in the molten pool area. Figure 12 shows the distribution diagram of powder velocity at different positions in the molten pool area, in which (a) is the laser DED process, and (b) is the hybrid process. It can be seen from the figure that the distribution range of forming powders spreads from about 1.5 mm in (a) to 3 mm in (b). For the influence of shot peening gas flow and shot peening particles, the distribution range of forming powder is slightly diffused. The center of the powder flow is shifted by about 1 mm, but it is still within 3 mm of the spot. The quantity of powder varies little. The velocity distribution of forming powder is more dispersed and the magnitude of velocity does not change significantly. Although only a small amount of shot peening gas flow goes to the forming area under the action of hot pressure, powder feeding gas and protective gas in the molten pool, it still has a certain influence on the forming process. In the subsequent experimental verification, the method of reducing the scanning interval was adopted to eliminate the influence of the shot peening gas flow, so as to ensure the quality of the samples formed by the hybrid process.

### 3.2. Experimental Validation

#### 3.2.1. Temperature Field Verification

**Figure 13:**
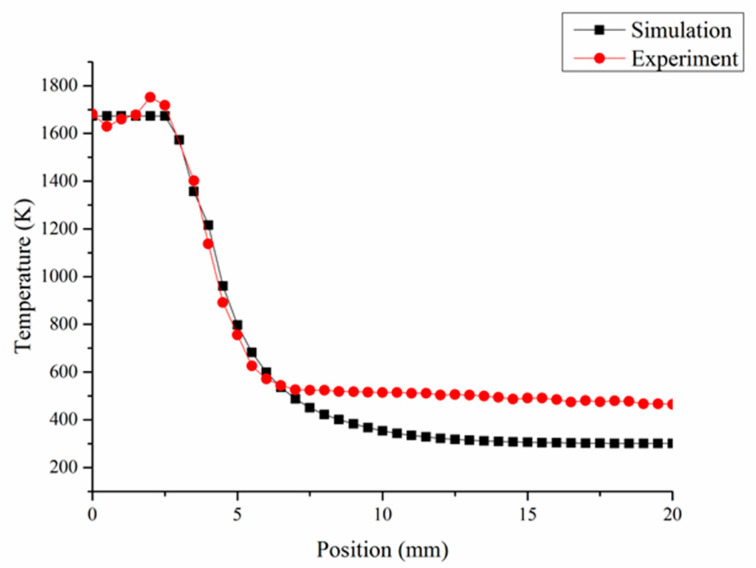
Temperature change curve near the molten pool.

Figure 13 shows the temperature change curve from the center of the molten pool to the laser-traveled region. In the figure, the experimental results of temperature field distribution and temperature gradient basically accord with the simulation results, but there are some shortcomings. The actual temperature in the molten pool fluctuates to some extent, and the actual temperature in the low temperature range of the hybrid process is higher than in the simulation results. This is because in the forming process, with the increase of the number of laser cladding layers, there will be a certain degree of thermal accumulation in effect—which was without consideration in the simulation model—which will change the temperature of the substrate before next layer’s formation, making the initial forming temperature higher than the simulation results. On the other hand, the movement of laser cladding nozzle will result in some heat remaining in the traveled area, which was also not included in the simulation. There was little influence on the high temperature range due to laser forming characteristic. Firstly, the temperature at the center of the molten pool was difficult to change, for enough laser power was used in the experiments. Secondly, laser DED is characterized by rapid cooling and solidification. A high temperature range may disappear in several seconds. Fortunately, both settings mainly influence the low temperature range (lower than stress relaxation temperature of austenitic stainless steels [28]) and the result does not affect the temperature range where the shot peening point is located, and the parameters of shot peening in the hybrid process can still be optimized effectively. The temperature at the shot peening point is about 571 K (298 ℃), and it is still in the stress relaxation zone of the material [28], which can have an effective shot peening effect.

#### 3.2.2. Gas Flow Interference Verification

**Figure 14:**
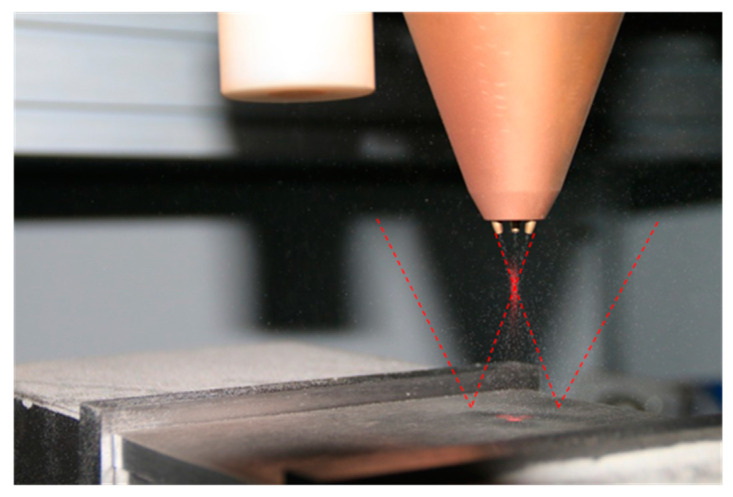
Powder trajectory before shot peening.

**Figure 15:**
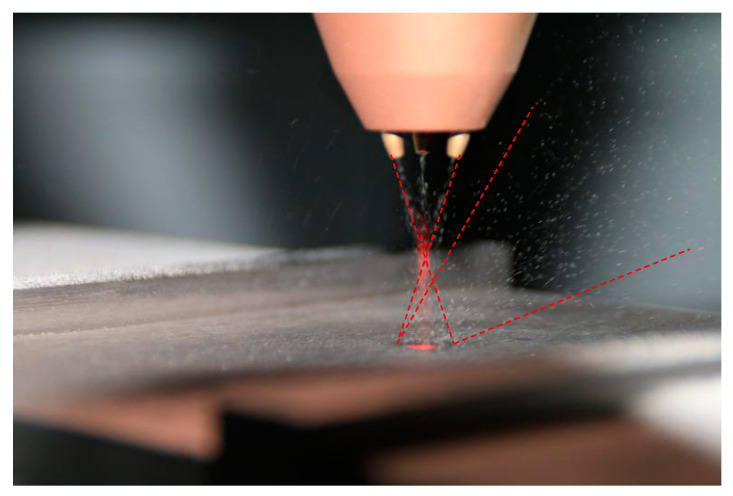
Powder trajectory when shot peening.

The laser cladding nozzle used in the experiment was a coaxial powder feeding type. The powder convergence of the laser cladding process before and after shot peening was observed respectively to verify the influence of the shot peening gas flow. Figure 14 and Figure 15 show the powder trajectory before shot peening and when shot peening in the hybrid process. When powder feeding without shot peening is carried out, the powders converge at the laser’s focal point well and are distributed symmetrically in space after colliding with the substrate. When both shot peening and powder feeding are carried out at the same time, the powder flow can still keep converging at the laser’s focal point, and the trajectory of forming powder hardly changed before the convergence. On right side of the space, there are many powder tracks, which are mainly shot peening particles bounced on the substrate, but a small part of them were forming powder that contacted the substrate at low speed. The powder is affected by shot peening gas flow and bounces back to the side. The forming quality of laser DED is mainly related to the trajectory of powder before convergence, and the hybrid process can be developed.

#### 3.2.3. Forming Sample Property

**Figure 16:**
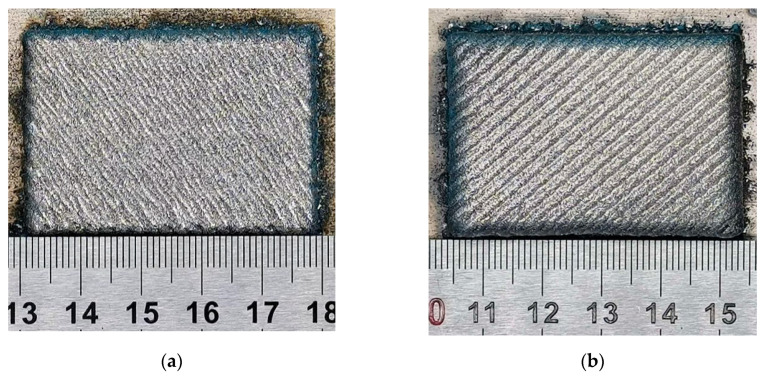
Forming samples under the optimized hybrid process. (**a**) Sample with peening and (**b**) sample without peening.

The forming samples under the optimized process are shown in Figure 16. Table 4 shows the properties of samples formed by the optimized hybrid process. After the optimization of the hybrid process, the waste heat generated in the laser DED process is utilized and the interference in the two processes is reduced, thus the hybrid process is realized. The density and stress of samples formed by the hybrid process were improved compared with those of cladding samples. The density increased from 7.075 to 7.700 g/cm^3^, an increase of 8.83%. The average residual stress on the surface of the sample formed by hybrid process changed from tensile stress to compressive stress, and the introduced compressive stress was 511.6 MPa, which is 2.26 times the tensile stress on the surface of the laser cladding sample, and has a good forming quality. In addition, due to the plastic deformation and grain refinement caused by shot peening, the hardness of the sample formed by the hybrid process has been improved from 407.4 to 423.5 HV, an increase of 3.95%. At the same time, the surface of the sample was eroded by the powder collision, which changed the roughness from 39.13 to 25.18 Ra. The oxide layer on the surface of the sample formed by hybrid process was removed, and it showed more metallic luster.

## 4. Conclusions

Based on the difficulties of the hybrid process of laser DED and shot peening, using a discrete phase model and fluid–solid coupling model in the CFD method, the hybrid process was simulated, and the shot peening point was optimized. The influences of the shot peening gas flow in the hybrid process on the temperature field and the powder convergence were studied, and experimental verification was carried out. The following conclusions are drawn:

1. When the shot peening angle is 45.6° and the shot peening point is located at 6 mm from the cladding point, the temperature is in the stress relaxation zone of the material. In the hybrid process, shot peening has a good strengthening effect, the density of the forming sample is increased by 8.83% and the introduced compressive stress is 2.26 times the tensile stress on the surface of the cladding sample.
2. In the hybrid process, shot peening gas flow changes the temperature field and presents a bidirectional cooling trend with the injection point as the center, which speeds up the reduction rate in the high temperature area and slows down the reduction rate in the low temperature area.
3. In the hybrid process, shot peening gas flow has little influence on the powder convergence, the range of powder trajectory is expanded and the center position of powder flow is shifted by 1 mm. The method of reducing the laser scanning interval is adopted to improve the forming quality in the experiment.
4. By experimental verification, it was found that the temperature variation rule and the powder trajectory basically confirm the simulation results. In the simulation, the influence of heat accumulation and the fluctuation of the molten pool temperature in the process were ignored, causing the low temperature value to be lower than the experimental value, but stress relaxation temperature at the injection position basically conformed to the simulation results. It still met the requirements of process optimization.

## Figures and Tables

**Table 1:** The thermal conductivity used in simulation.

**T/K**	293	373	473	573	673	773	873	973	1073	1173	1273	1373	1473
**Thermal Conductivity/** **W/(m·K)**	16.6	17.2	18.0	18.7	19.4	20.1	20.8	22.2	23.4	24.8	26.1	27.5	28.9

**Table 2:** The initial boundary conditions of the model.

Parameter	Value
Peening inlet pressure	0.4 MPa
Powder feeding inlet pressure	0.3 MPa
Shielding gas inlet pressure	0.3 MPa
Outlet	0 MPa
Cladding Temperature	1673 K
Substrate Wall	reflect/couple

**Table 3:** Components of Fe–Cr–Ni–B–Si alloy powder.

Element	Cr	Ni	B	Si	Fe
**Mass fraction**	15	10	1	1	Bal.

**Table 4:** The properties of the samples.

Process	Density (g/cm^3^)	Stress (MPa)	Hardness (HV)	Roughness (Ra)
Laser DED	7.075	226.7	407.4	39.13
Hybrid process	7.700	−284.9	423.5	25.18
